# Supplementary material for: Reporting of 3Rs Approaches in Preclinical Animal Experimental Studies—A Nationwide Study
Source: Animals (Basel). 2023 Sep 23;13(19):3005. doi: 10.3390/ani13193005 (PMC10571812; doi:10.3390/ani13193005)
Supplement: Supplementary file 1 [file animals-13-03005-s001.zip › File S1.pdf]

Study protocol
- modified for references

Preliminary title: Investigation and analyses of internal validity in Danish preclinical research

Contact person:
Birgitte S. Kousholt, DVM, PhD
Department of Clinical Medicine, Aarhus University,
Palle Juul Jensens Boulevard 99, 8200 Aarhus N, Aarhus, Denmark
birgitte.kousholt@clin.au.dk

Protocol registration date and location: 2018-11-07 www.syrcle.nl

1. BACKGROUND

Poor reproducibility in biomedical research is a source of wasteful use of animals. *Reduction* of animals can be achieved by an overall improvement of the internal validity of experiments. Internal validity refers to the extent to which the design and conduct of an experiment eliminates the risk of introducing bias. Several measures to strengthen internal validity exist, such as blinding, randomisation and proper sample size calculation. By limiting bias, any

influence that will jeopardize the true result are minimised. Thorough and accurate guidelines on planning and reporting experiments such as PREPARE [9] and ARRIVE [15] are readily available. Although it may seem an easy task to comply with these, a 2010 investigation provides concern that this is not at all occurring – at least not among leading universities in the UK. A quality assessment (funded by NC3Rs) of research from UK universities showed poor implementation of risk of bias (RoB) limiting practices such as sample size calculation, randomization and blinding. We speculate that this is also the case in Denmark as in general, existing guidelines are not having the desired impact [16,17]. This is interesting from a 3R perspective because as stated by Russel & Burch in 1959 (on the importance of designing and analysing experiments): "Every time any particle of statistical method is properly used, fewer animals are employed than would otherwise have been necessary"[1]. This means that poor internal validity of animal experiments leads to waste of a great number of animals.

A procedure to indirectly measure internal validity is by use of a quality assessment tool well-known from the evidence based systematic review methodology. The tool investigates methodological reporting and has recently been adjusted for use in preclinical animal experiments. Application on pilot data from an internal study of published animal research from Aarhus University suggests that the quality of methodological reporting is very poor. Assuming this trend is similar across all Danish research institutions indicates a nationwide neglect of easily applicable research methods, which directly will contribute to reduction. Evidence-based proof is nevertheless missing. Meta-research is necessary, and will open the way for advancement of reduction in Denmark [18]

1.1 Research questions

1. What is the current prevalence of risk of bias in experimental animal research?
2. How is it compared to the prevalence of risk of bias in experimental animal research from 2009, the year before publication of the ARRIVE guidelines?
3. What is the prevalence of risk of bias compared among five Danish universities and how do they compare to the national average?

2. METHODS

2.1 Study protocol

A pre-specified systematic review protocol for animal intervention studies offered by the SYstematic Review Centre for Laboratory animal Experimentation (SYRCLE) is modified for the aims of the study.

2.2 Condition of interest

The risk of bias in animal experimental research where one or more authors has/have an affiliation to one of five Danish universities will be investigated.

2.3 Population studied

This study will include papers reporting animal experiments from Aarhus University (AU), Aalborg University (AAU), University of Copenhagen (UCPH), University of Southern Denmark (SDU) or Technical University of Denmark (DTU).

2.4 Intervention

The study will include any animal experimental study involving non-human vertebrates fulfilling inclusion criteria.

2.5 Outcome measures

The outcome of the study is the reporting of risk of bias items given by:

1. Sample size calculation and statement of sample size (other bias)
2. Randomization (selection bias)
3. Blinding to treatment/intervention and blinding of outcome assessment (performance and detection bias)
4. Statement of the number of animals at the end of the study and description of excluded animals or dropouts (attrition bias)

The abovementioned types of bias are based on the Landis 4. Also included will be other important risks of introducing a bias such as:

5. Health status of the animals at the beginning of experiment
6. Statement of whether the study is conducted in compliance with animal welfare requirements

7. Statements of whether any specific measures have been taken to implement the 3Rs, replacement, reduction or refinement
8. Statement of a declaration of conflict of interest

2.6 Search and study identification

A systematic literature search will be performed in collaboration with a library information specialist. All potentially relevant studies will be retrieved by the use of a modified, comprehensive search strategy devised by SYRCLE. All in vivo studies performed in non-human vertebrates with an author affiliated to at least one of five Danish universities of interest will be retrieved. Additional filters restricting the results to include only publications from 2009 and 2018, respectively, will be added.

2.7 Study selection

The search results from different databases will be pooled in two separate libraries in the reference manager programme and duplicates will be removed (2009 and 2018, respectively). Study eligibility screening will be a two-phase process:
1) Pre-screening based on title and abstract
2) Full text screening of papers that cannot be included or excluded based solely on title and abstract

Two reviewers will independently screen titles and abstracts, after which full-text articles meeting the inclusion criteria will be identified and reviewed. Where a decision cannot be reached, an additional reviewer will read the full-text articles and a decision will be made. The following inclusion and exclusion criteria will be used to assess study eligibility for the present systematic review:
Inclusion criteria are: manuscripts describing research in life sciences (more specifically in vivo research containing at least one non-human animal intervention experiment in vertebrates), combined in vitro and in vivo research containing at least one animal intervention experiment as described above, manuscripts published during 2018 (1st

aim, 1st priority) or during 2009 (2nd aim, 2nd priority), and affiliated to one of five universities of interest.

Exclusion criteria are: manuscripts containing or characterized by the following: exploratory studies without hypothesis testing statistical analyses and interventions, not English, doubles, farm animal target, wild animal target, environmental investigations, human studies, not in vivo, not primary paper (review, abstract, proceedings, supplement, image, PhD thesis etc.)

In the pre-screening selection phase exclusion criteria are: farm or wild animal target, environmental investigations, human studies, not in vivo, not primary paper.

In the full-text screening phase exclusion criteria are: farm or wild animal target, environmental investigations, exploratory studies without hypothesis testing statistical analyses, human studies, not in vivo, not primary paper.

2.8 Data extraction and analysis

Publications will be assessed by two independent reviewers, each blinded to the assessment of the other. Reviewers will examine the full text of the articles, including figures and tables in addition to supplemental information. The publications will be assessed for reporting of different types of bias related to selection bias, performance bias, detection bias and attrition bias. Some types of biases are subdivided to make assessment more manageable. Furthermore, studies will be assessed for consideration of compliance with legislation and the 3Rs (reported measures taken to include replacement, reduction or refinement in the study setup and for compliance with animal welfare requirements). For each included study, each item will be scored as dichotomous (yes/no reporting assessments). The term unclear may be used to specify 'not applicable' to the design of the study. Discrepancies will be resolved by discussion until consensus, or if that is not possible, by a third reviewer blinded to the assessment of the first two.

For each risk of bias item the prevalence of reporting and 95% confidence intervals will be reported. Two-sample proportion test will be performed to assess any

differences in reporting prevalence. The prevalence of reporting of each risk of bias item will be calculated for each university and compared to the national average as well as to the reporting prevalence of the other universities.

Study updates and protocol amendments

The following updates and changes have been made to this version of the protocol.

In section 1.1 we have specified our research questions to:

1. What is the current *prevalence of reporting* in experimental animal research?

2. How is it compared to the *prevalence of reporting* in experimental animal research from 2009, the year before publication of the ARRIVE guidelines?

Research question three was not investigated further due to the experimental design and the procedure for random sampling.

In sections 1.2 we convey *the quality of methodological reporting* in animal experimental research with one or more authors affiliated to a Danish university. Application on pilot data from an internal study of published animal research from Aarhus University suggests that the quality of methodological reporting is very poor. Taking this into account, we chose to modify our proposed study approach. We assessed publications for the quality of methodological reporting of selected items safeguarding against bias by first evaluating the overall reporting status. Thereafter, we provided additional details by emphasizing the level of detail given for each of the reported items.

In section 2.2 the condition of interest is to convey *the methodological reporting*

In section 2.8 we decided not to use the score "unclear" in this study. We decided to code all reported information to convey the level of detail in the information given for reported items.

The result of the reporting of outcome measure 1-5 and 8 investigated in this study will be published separately.

REFERENCES

9. Smith, A.J., et al., *PREPARE: guidelines for planning animal research and testing.* Lab Anim, 2018. **52**(2): p. 135-141.

15. Ting, K.H., C.L. Hill, and S.L. Whittle, *Quality of reporting of interventional animal studies in rheumatology: a systematic review using the ARRIVE guidelines.* Int J Rheum Dis, 2015. **18**(5): p. 488-94.

16. Kilkenny, C., et al., *Improving bioscience research reporting: the ARRIVE guidelines for reporting animal research.* PLoS Biol, 2010. **8**(6): p. e1000412.

17. Baker, D., et al., *Two years later: journals are not yet enforcing the ARRIVE guidelines on reporting standards for pre-clinical animal studies.* PLoS Biol, 2014. **12**(1): p. e1001756.

1. Russel, W.M.S. and R.L. Burch, *The principles of humane experimental technique*. 1959.

18. Avey, M.T., N. Fenwick, and G. Griffin, *The use of systematic reviews and reporting guidelines to advance the implementation of the 3Rs.* J Am Assoc Lab Anim Sci, 2015. **54**(2): p. 153-62.
