# Supplementary material for: Reporting of 3Rs Approaches in Preclinical Animal Experimental Studies—A Nationwide Study
Source: Animals (Basel). 2023 Sep 23;13(19):3005. doi: 10.3390/ani13193005 (PMC10571812; doi:10.3390/ani13193005)
Supplement: Supplementary file 1 [file animals-13-03005-s001.zip › Table S1.pdf]

Table S1. Data extraction form to facilitate assessment of reporting compliance with legislation on animal experimentation and the 3Rs.

The form consists of five items with questions to facilitate assessment of reporting. Each item has only one answer option, which could be “Yes” or “No”. Items are scored “Yes” if the item is reported or “No” when there is no reporting of the item. In case of difficult scoring decisions (e.g., the item is partially reported) the study is scored as “Yes” and notes are saved to provide clarification about the item. Thereafter, the reporting quality is discriminated by a numerical score. A score of 3, 2, 1, or 0 is given according to the quality of information pertaining to each question.

**Item**	**Question**	**Score**			
		**“No” (Not reported)**	**“Yes” (Reported)**		
		**0**	**1**	**2**	**3**
Compliance with legislation on animal experimentation	Identification of committee approving the animal experiments?	Not reported	Reported	-	-
The 3Rs	Is there a statement about any specific measures taken to implement the 3Rs?	Not reported	Reported with no specific method	Reported with a specific method	Reported to be in compliance with the 3Rs or the ARRIVE guidelines
Replacement	Is there a statement about any specific measures taken to implement the principle of replacement?	Not reported	Reported with no specific method	Reported with a specific method	-
Reduction	Is there a statement about any specific measures taken to implement the principle of reduction?	Not reported	Reported with no specific method	Reported with a specific method	-

Refinement	Is there a statement about any specific measures taken to implement the principle of refinement?	Not reported	Reported with no specific method	Reported with a specific method	-
