# Supplementary material for: Reporting of 3Rs Approaches in Preclinical Animal Experimental Studies—A Nationwide Study
Source: Animals (Basel). 2023 Sep 23;13(19):3005. doi: 10.3390/ani13193005 (PMC10571812; doi:10.3390/ani13193005)
Supplement: Supplementary file 1 [file animals-13-03005-s001.zip › Table S2.pdf]

Table S2. Criteria for the assessment of reporting compliance with legislation on animal experimentation and the 3Rs[1] - examples.

Compliance with legislation on animal experimentation

Criteria for a judgement of "Yes" (Reported)	Reported that the study is conducted in compliance with legislation on animal experimentation	Compliance with legislation on animal experimentation mentioned	Quote: "E*xperiments were approved by the Danish Animal Experiments Inspectorate and conducted in accordance to the ethical legislation for the care and use of experimental animals."*

The 3Rs

Criteria for a judgement of "Yes" (Reported)	Reported with no specific method to implement the 3Rs	Reported 3R sentences of replacement, reduction, and refinement with no method to implement one or more of the 3Rs	"Quote: *"All efforts were made to minimize both the suffering and the number of animals."*
	Reported with a specific method to implement the 3Rs	Reported 3R sentences of replacement, reduction, and refinement with a specific method to implement one or more of the 3Rs	Quote: *"Following the principles of replacement, reduction, and refinement for animal studies, we used pigs that were also part of a previously published study."*
	Reported to be in compliance with the 3Rs and/or the ARRIVE guidelines	Compliance with the 3Rs and/or the ARRIVE guidelines mentioned	Quote: *"The experimental protocol was designed taking into account the Russel and Burch 3R's principle.* Quote: *"All experiments were reported in compliance with the ARRIVE guidelines (Animal Research: Reporting in Vivo Experiments)"*

Replacement

Criteria for a judgement of "Yes" (Reported)	Reported without a specific method to implement replacement	Replacement mentioned with no specific method to implement replacement	Quote: None mentioned
	Reported with a specific method to implement replacement	Replacement mentioned with a specific method to implement replacement	Quote: None mentioned

Reduction

Criteria for a judgement of "Yes" (Reported)	Reported *without* a specific method to implement reduction	Reduction mentioned with no specific method to implement reduction	Quote*: "Every effort was made to minimize the number of mice used..."*
	Reported *with* a specific method to implement reduction	Reduction mentioned with a specific method to implement reduction	Quote: *"...to minimize the number of mice used for experiments, we combined the cytokine assay with a glymphatic assay for clearance.*

Refinement

Criteria for a judgement of "Yes" (Reported)	Reported without a specific method to implement refinement	Refinement mentioned with no specific method to implement refinement	Quote: *"All efforts were made to minimize animal suffering."*
	Reported with a specific method to implement refinement	Refinement mentioned with a specific method to implement refinement	Quote: *"To minimize stress, animals were habituated…"*

[1] Modified from Cochrane Handbook for Systematic Reviews of Interventions, version 5.2.0 (updated June 2017), Cochrane, 2017
